# Comparing PI3K/Akt Inhibitors Used in Ovarian Cancer Treatment

**DOI:** 10.3389/fphar.2020.00206

**Published:** 2020-03-03

**Authors:** Yi-Hui Wu, Yu-Fang Huang, Chien-Chin Chen, Chia-Yen Huang, Cheng-Yang Chou

**Affiliations:** ^1^Department of Medical Research, Chi Mei Medical Center, Liouying, Taiwan; ^2^Department of Obstetrics and Gynecology, National Cheng Kung University Hospital, College of Medicine, National Cheng Kung University, Tainan, Taiwan; ^3^Department of Pathology, Ditmanson Medical Foundation Chia-Yi Christian Hospital, Chiayi, Taiwan; ^4^Department of Cosmetic Science, Chia Nan University of Pharmacy and Science, Tainan, Taiwan; ^5^Department of Biological Science and Technology, College of Biological Science and Technology, National Chiao Tung University, Hsinchu, Taiwan; ^6^Gynecologic Cancer Center, Department of Obstetrics and Gynecology, Cathay General Hospital, Taipei, Taiwan; ^7^School of Medicine, Fu Jen Catholic University, New Taipei City, Taiwan

**Keywords:** epithelial ovarian carcinoma, chemoresistance, PI3K inhibitor, Akt inhibitor, cisplatin, paclitaxel

## Abstract

Epithelial ovarian carcinoma (EOC) is the most lethal gynecological malignancy. Herein, we sought to determine the efficacy of phosphoinositide 3-kinase (PI3K)/Akt inhibition using three AZD compounds in a NOD-SCID xenograft mouse model and Akt regulation in a panel of eight ovarian cancer cell lines. Elevated Akt phosphorylation on Ser473 but not on Thr308 in cancerous tissues correlated with short progression-free survival (PFS), overall survival (OS), and death. AZD8835 and AZD8186 inhibited Akt phosphorylation while AZD5363 augmented its phosphorylation on Ser473. To add, all compounds inhibited the Akt downstream effectors 4E-BP1 and p70S6 kinase. AZD8835 and AZD5363 sensitized chemoresistant ovarian cancer cells to cisplatin and paclitaxel treatment. Only AZD5363 could inhibit COL11A1 mRNA and promoter activity, which are important factors in Akt regulation and chemoresistance in ovarian cancer. By using a mouse xenograft model, AZD8835 and AZD5363, but not AZD8186, caused a significant reduction in tumor formation. AZD compounds did not change the mRNA expression of BRCA1/BRCA in ovarian cancer cells, but AZD8835 inhibited BRCA1/BRCA2 mRNA expression and p-ERK protein expression in OVCAR-8 cells with the *KRAS* mutation. This study highlights the importance of PI3K/Akt in ovarian tumor progression and chemoresistance and the potential application of AZD compounds, especially AZD8835 and AZD5363, as therapeutic agents for the treatment of ovarian cancer.

## Introduction

Epithelial ovarian carcinoma (EOC) is the most lethal gynecological malignancy (Siegel et al., 2012). Most patients with EOC are diagnosed at an advanced stage and although most initially respond to cytoreductive surgery and platinum-based chemotherapies, many EOC patients eventually relapse, develop chemoresistant tumors, and die because of the disease (Ozols, 2006; Waldmann et al., 2013). As the empirical incorporation of additional cytotoxic agents against ovarian cancer does not improve prognosis (Bookman et al., 2009), understanding the key signaling pathway responsible for chemoresistance and disease progression is required to develop new treatment strategies.

The phosphoinositide 3-kinase (PI3K)/protein kinase B (Akt) is a critical regulator of many cellular processes, including metabolism, growth, and anti-apoptosis, and is frequently dysregulated in human cancers (Thorpe et al., 2015). The PI3K family consists of three distinct classes (I–III), each with different structures and substrate specificities. Class I PI3Ks are further divided into subclasses IA and IB, and IA subclass is the most frequently activated in cancer (Thorpe et al., 2015). Class IA molecules are heterodimers consisting of a p110 catalytic subunit complexed with a p85 regulatory subunit. There are three isoforms of the p110 catalytic subunit (α, β, and δ), encoded by *PIK3CA*, *PIK3CB*, and *PIK3CD* genes, respectively. Multiple mechanisms may be involved in PI3K mutation and aberrant activation in human cancers (Samuels and Ericson, 2006; Engelman, 2009; Jaiswal et al., 2009; Vanhaesebroeck et al., 2010; Cheung et al., 2011; Cancer Genome Atlas Research Network, 2012; Walter et al., 2018). PI3K activation leads to the Akt activation and subsequent activation of downstream effectors, such as mTOR and others (Mundi et al., 2016). Akt activation involves the phosphorylation of two residues: threonine 308 (T308) in the activation loop of the kinase by the protein kinase, Akt-3-phosphoinositide-dependent kinase 1 (PDK1), and serine 473 (S473) in the hydrophobic motif of the mTORC2 complex (Alessi et al., 1997; Vanhaesebroeck and Alessi, 2000; Sarbassov et al., 2005).

In addition to the aforementioned cellular processes in cancer, Akt activation is also associated with resistance to both chemotherapeutic agents and target agents (Clark et al., 2002). Hence, Akt activity may be a potential therapeutic target and biomarker (Navid and Gerber, 2012; Thorpe et al., 2015; Mundi et al., 2016); however, no significant results have been reported from clinical trials with PI3K/Akt inhibitor monotherapy in solid tumors (Hanker et al., 2019). In addition, the use of PI3K/Akt inhibitors in ovarian cancer is scarcely investigated. Our previous report (Wu et al., 2019) indicated that Akt inhibitors, namely, SC66 and MK-2206, might inhibit Akt signaling through various mechanisms, and suggested that the evaluation of PI3K/Akt/mTOR inhibitors is critical to confirm the sensitivity patterns observed in preclinical studies prior to clinical use. The aim of this study is to determine the significance of PI3K/Akt activation in ovarian cancer by examining Akt expression in tumor specimens, and the effects of PI3K/Akt inhibition using three AZD compounds, namely, AZD5363, AZD8835, and AZD8186 which are small molecule drugs. AZD5363, a potent pan-Akt kinase inhibitor, inhibits the growth of a wide range of human tumor xenografts, and is used as monotherapy or in combination with HER2 inhibitors or docetaxel in a breast cancer model (Davies et al., 2012). AZD8835 is an isoform-selective inhibitor of PI3Kα and PI3Kδ that preferentially inhibits cells growth with mutant PIK3CA status such as in estrogen receptor-positive (ER^+^) breast cancer cell lines and xenografts (Hudson et al., 2016). AZD8186, a PI3Kβ and PI3Kδ inhibitor, inhibits the growth of various tumor cell lines, as well as effectively inhibits tumor growth in prostate and triple-negative breast cancer models (Hancox et al., 2015).

## Materials and Methods

### Akt Expression and Clinical Data Obtained From the Cancer Proteome Atlas (TCPA) and the Cancer Genome Atlas (TCGA) Databases

Phospho-Akt (p-Akt) on Ser473 (S473), p-Akt on Thr308 (T308), and Akt protein expression in cancerous tissues and survival analysis of ovarian cancer patients were directly obtained from The Cancer Proteome Atlas (TCPA) database^1^ (Li et al., 2013, 2017). In the database, the aforementioned protein levels were determined, and samples were divided into low- and high-expression groups, according to the median value of protein expression.

Clinical data for these patients were collected from The Cancer Genome Atlas (TCGA) database^2^ in May 2019. The clinicopathological parameters were age, International Federation of Gynecology and Obstetrics (FIGO) stage, size of residual tumor nodules after surgery, primary therapy outcome, and survival status.

### Patient Population

Patients with stage I–IV EOC, tubal cancer, or primary peritoneal cancer, according to the FIGO cancer staging system, who underwent staging surgery or cytoreduction at the National Cheng Kung University Hospital between 2000 and 2010 were enrolled in the study. Patients were followed after treatment, with 31 May 2019 as the date of the latest record retrieval. Both progression-free survival (PFS) and overall survival (OS) were calculated from the diagnosis. Medical records and pathology slides for these patients were the source of information for clinical characteristics, pathological diagnoses, progression-free intervals (PFIs), and outcomes. The investigation was approved by the National Cheng Kung University Hospital Institutional Review Board (A-ER-105-017) and written consent from each patient was obtained. The study methodologies in accordance with the standards set by the Declaration of Helsinki.

### Evaluation of p-Akt Levels by Immunohistochemistry (IHC)

Ovarian cancer tissue sections were prepared as previously described (Wu et al., 2015). Formalin-fixed paraffin embedded tissue sections were deparaffinized and stained for p-Akt protein using a standard automated immunohistochemistry (IHC) slide staining system (BOND-MAX Autostainer, Leica Microsystems, United Kingdom) after antigen retrieval in EDTA buffer, pH 9.0, for 20 min. The anti-p-Akt (S473) primary antibody (4060S; Cell Signaling Technology, Danvers, MA, United States) at a dilution of 1:100 and the anti-p-Akt (T308) primary antibody (GeneTex, Irvine, CA, United States) at a dilution of 1:100 were then added. Negative controls were treated with phosphate-buffered saline (PBS). High-expressing p-Akt (S473) human lung carcinoma and p-Akt (T308) breast carcinoma were used as positive controls. The investigator who evaluated these experiments (C-CC, a gynecologic pathologist) was blinded to the patient clinical outcome data. Staining intensity was categorized as follows: negative, weak, moderate, and strong staining (grade 0, 1, 2, and 3, respectively). A grade 0–2 in staining intensity was designated as low expression whereas a grade 3 was considered as high expression (Supplementary Figure S1).

### Cells and Media

The human ovarian cancer cell lines, A2780 and A2780CP70, were obtained from the American Type Culture Collection (ATCC, Manassas, VA, United States). The HAC-2 cell line was obtained from the Japanese Collection of Research Bioresources (JCRB) Cell Bank (Osaka, Japan). The OVCAR-3, OVCAR-4, and OVCAR-8 cell lines were purchased through the National Cancer Institute DTP tumor repository program (Frederick, MD, United States). The ES-2 cell line was purchased from the Bioresource Collection and Research Center of the Food Industry Research and Development Institute (Hsinchu, Taiwan). Cisplatin-resistant strains of ES-2 (ES-2/CP) were developed in our laboratory as described previously (Wu et al., 2019). The cell lines A2780, A2780CP70, OVCAR-3, OVCAR-4, and OVCA-8 were grown in RPMI-1640 medium supplemented with 10% fetal bovine serum (FBS). HAC-2 cells were grown in Minimal Essential Medium (MEM) supplemented with 15% FBS. ES-2 and ES-2/CP cells were grown in Mycos 5A medium supplemented with 10% FBS. All cells were grown at 37°C in a 5% carbon dioxide atmosphere. Cells were cultured and stored according to the supplier’s instructions and used between passages 5 and 20. Once thawed, the cell lines were routinely authenticated every ∼6 months, with the last cell testing performed in March 2019 by cell morphology monitoring, growth curve analysis, species verification by iso-enzymology and karyotyping, identity verification using short tandem repeat-profiling analysis, and contamination checks.

### Western Blotting

Proteins were extracted and equal amounts were separated by 8–15% sodium dodecyl sulfate-polyacrylamide gel electrophoresis (SDS-PAGE), as described previously (Wu et al., 2014).

### Antibodies and Reagents

Antibodies specific to PDK1 (3062), phospho-Akt (Thr308, 4056), phospho-Akt (Ser473, 9271), Akt (9272), phospho-p70S6K (9205), P70S6K (9202), phospho-4EBP1 (9451), 4EBP1 (9452), phosphor-C-Raf (Ser259, 9421), ERK (9102), phospho-ERK (Thr202/Tyr204, 9101), mouse IgG (7076), and rabbit IgG (7074) were obtained from Cell Signaling Technology. Anti-β-actin (sc-47778) antibody was purchased from Santa Cruz Biotechnology. Antibody against COL11A1 (GTX55142) was obtained from GeneTex (Irvine, CA, United States). Antibody against KRAS (ab180772) was obtained from Abcam (Cambridge, United Kingdom). The PI3K inhibitors (AZD8186 and AZD8835) and Akt inhibitor (AZD5363) were synthesized at AstraZeneca (Cambridge, United Kingdom). Cisplatin (Fresenius Kabi Oncology, Ltd.) and paclitaxel (Corden Pharma Latina S.P.A.) were provided by the Cancer Center of National Cheng Kung University Hospital.

### Calculation of Half-Maximal Inhibitory Concentration (IC_50_) and Combination Index (CI) Analysis

Cisplatin (10 mM) and paclitaxel (10 mM) were dissolved in distilled water whereas AZD5363, AZD8186, and AZD8835 (10 mM) stock solutions were prepared in dimethylsulfoxide, stored at −20°C, and diluted to a final dimethylsulfoxide concentration of less than 0.5%. When the experiment was carried out, the culture medium was used for drug dilution. Cells (1 × 10^4^) were exposed to varying concentrations of cisplatin (0–32 μM), paclitaxel (0–64 μM), and three AZD compounds (0–100 μM) for 48 h. The *in vitro* cytotoxic effects of these treatments were determined using a 3-(4,5-dimethylthiazol-2-yl)-2,5-diphenyltetrazolium bromide (MTT) assay (at 570 nm) with a final MTT concentration of 0.125 mg/mL, and cell viability was expressed as a percentage of the viability of control cells (% of control). IC_50_ values were determined from a dose-response curve of percent growth inhibition against test concentrations. For combination treatment, cells were co-treated with AZD compound and different concentrations of cisplatin (0–32 μM) or paclitaxel (0–64 μM) for 48 h. Combination index (CI) analysis is the most common method used to evaluate the nature of drug interactions in combination chemotherapy and provide useful quantitative information (Chou and Talalay, 1984). CI is a numerical value calculated with the following formula: CI = C_A,X_/IC_X,A_ + C_B,X_/IC_X,B_, where C_A,X_ and C_B,X_ represent the concentrations of drug A and drug B when used in combination to achieve x% drug effect. IC_X,A_ and IC_X,B_ represent the concentrations required for individual monotherapy to achieve the same x% effect. CI < 1 indicates synergy, CI = 1 demonstrates an additive effect, and CI > 1 represents antagonism (Chou and Talalay, 1984).

### Quantitative Reverse Transcriptase PCR (RT-PCR)

Total RNA (5 μg) was used as the template in the cDNA-synthesis reactions with random primers and Superscript III reverse transcriptase (Applied Biosystems). The resultant cDNAs were used (at a 1:20 dilution) to detect the level of endogenous *BRCA1*, *BRCA2*, and *PD-L1* mRNA expression by quantitative PCR (qPCR). Accurate quantitation was achieved using standard curves generated by serially diluting a known quantity of RNA from an *in vitro* transcription reaction and performing TaqMan qPCR with the dilution along with the cell samples. Quantitative analysis of mRNA expression was performed with the StepOne^TM^ Real-Time PCR System (ABI). The primers and TaqMan probes used for the analyses were designed using the manufacturer’s software, Primer Express. The following primers were used: BRCA1 (HS01556193), BRCA2 (HS00609073), and GAPDH (HS99999905). No-reverse-transcription (no-RT) control reactions were performed with 100 ng of total RNA from each individual sample as a template to ensure that amplification was not caused by DNA contamination. No signal was detected in the no-RT controls. Target gene mRNA expression was assessed by real-time RT-PCR. The reference gene *GAPDH* was used as the internal control for RNA quality. All quantitative analyses were performed in duplicate to assess the consistency of the results. The relative expression levels of the target gene, normalized to *GAPDH* expression, were calculated as ΔC_t_ = C_t_ (target) – C_t_ (GAPDH). The ratio of the number of copies of the target gene mRNA to the number of copies of *GAPDH* was then calculated as 2^–Ct^ × K (K = 10^6^, a constant).

### Plasmid Construction and Site-Directed Mutagenesis

The *COL11A1* PCR product was cloned into the *Kpn*I and *Xho*I sites of a pGL4 vector. The resultant construct was confirmed by DNA sequencing. *COL11A1* promoter deletion constructs COL11A1–541/ + 1, COL11A1−541/−203, and COL11A1–202/ + 1 were similarly generated using a COL11A1–541/ + 1 construct as a template, as described previously (Wu et al., 2014).

### Luciferase Reporter Assays

Luciferase assays were performed 48 h after transfection, using a Dual-Luciferase Reporter Assay System (Promega). Normalized luciferase activity was reported as the ratio of luciferase activity to β-galactosidase activity, as described previously (Wu et al., 2014).

### Orthotopic Xenograft Animal Model

Our study complied with the National Centre for the Replacement, Refinement, and Reduction of Animals in Research, and was approved by the Institutional Animal Care and Use Committee of National Cheng Kung University. A human EOC xenograft model was established in 6-week-old female NOD/SCID mice (NOD.CB17-Prkdc Scid/NcrCrl) supplied by the Animal Center at National Cheng Kung University. This study followed the Animal Research: Reporting of *In Vivo* Experiments guidelines and the National Institutes of Health guide for the care and use of Laboratory animals (NIH Publications No. 8023, revised 1978). The study was conducted with female mice only as EOC occurs in females. The effect of sex on overall sample size was ruled out without influences. Animals were housed under pathogen-free conditions in groups of five per cage and allowed to acclimate for 1–2 weeks. Mice were fed a diet of animal chow and water throughout the experiment. As ES-2-derived cell lines are the only cell lines that can form ovarian tumors and ascites in our orthotopic animal model, they were selected for used in the current study. Mice were implanted intrabursally (at the surface of bilateral ovaries) with 1 × 10^6^ ES2 or ES2/CP cells (100 μL). To determine tumor volume by external caliper, the greatest longitudinal diameter (length) and transverse diameter (width) were measured (Tomayko and Reynolds, 1989). Tumor volume based on caliper measurements was calculated (mm) as length × width × height × 0.52. Animal studies that involved tumor volume measurement were usually performed with one control per experimental subject. Number of mice required to assess a 30% decrease in tumor volume was estimated to be at least three for each of the control and experimental groups, with power and type I error of 0.8 and 0.05, respectively. Mice were randomly assigned to one of the AZD treatment groups (*n* = 4/group). Animals in each group orally received an AZD drug 5 days after tumor implantation. AZD8835, AZD8186, and AZD5363 were generally formulated as a suspension in HPMC/Tween. The doses of the three AZD compounds used were determined according to the previous reports: (a) 50 or 100 mg/kg AZD8835 once daily, 2 days on: 5 days off (Hudson et al., 2016); (b) 25 or 50 mg/kg AZD8186 twice daily (0 and 6–8 h), 5 days:2 days off (Hancox et al., 2015); (c) 75 or 150 mg/kg AZD5363 once daily, 4 days on:3 days off (Davies et al., 2012). Tumor growth, tumor imaging, and body weights were determined as described previously (Wu et al., 2019). After 21 days, mice were sacrificed using CO_2_ inhalation and the xenograft tumor tissues were excised and measured. Tumors were removed, weighed, fixed in 10% formalin, embedded in paraffin, and sectioned (4 μm) for histopathology and IHC. Paraffin sections were stained with hematoxylin and eosin. An investigator (C-CC, a gynecologic pathologist) was responsible for interpreting the extent of cancer involvement in each organ. The anti-p-Akt (S473) primary antibodies (4060, Cell Signaling Technology) were applied, while negative controls were treated with PBS. Highly expressing p-Akt-positive human lung carcinoma was used as positive controls. Staining intensity was scored as negative, weak, moderate, and strong staining.

### Statistical Analysis

Data were analyzed using the Statistical Package for the Social Sciences software program, version 17.0 (SPSS Inc., Chicago, IL, United States). Interval variables are presented as mean ± SEM. Differences between groups were determined by Mann–Whitney *U*-test. Frequency distributions between categorical variables were compared using Pearson’s chi-squared test and Fisher’s exact test. Survival was estimated using the Kaplan–Meier method and results were compared by log-rank tests. *P*-values < 0.05 (two-sided) indicated statistical significance.

## Results

### Akt Expression and Clinical Outcome of EOC Patients Obtained From TCPA and TCGA Databases

The expression level of p-Akt (S473) was significantly high in cancerous tissues of patients with advanced FIGO stage (*p* = 0.014), residual tumor nodules ≥ 1 cm (*p* = 0.004), and unfavorable primary therapy outcome (stable disease and progressive disease, *p* < 10^–5^; Supplementary Table S1). In contrast, the expression level of p-Akt (T308) did not differ among these clinical parameters. Nonetheless, the expression level of Akt was only significantly high in patients with advanced FIGO stage (*p* = 0.01; Supplementary Table S1).

**FIGURE 1:**
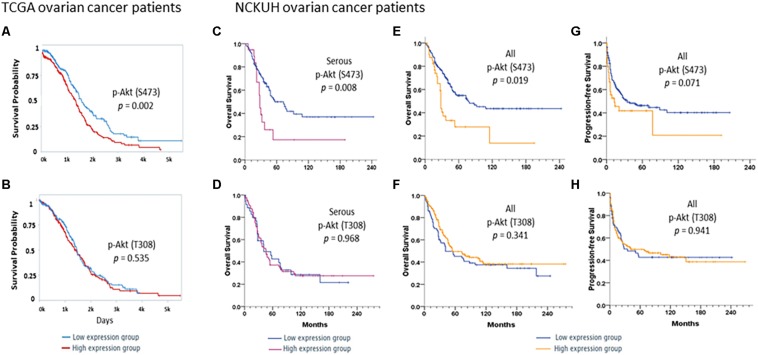
Survivals in TCGA ovarian cancer patients. Kaplan–Meier curves stratified by low and high protein expression of **(A)** p-Akt (S473) and **(B)** p-Akt (T308) by reverse-phase protein array in TCGA ovarian cancer patients with serous histology (*n* = 406). A log-rank test was also performed. Patients were divided into low (*n* = 203) and high (*n* = 203) expression groups based on the expression levels of these proteins. Survivals in NCKUH ovarian cancer patients. Kaplan–Meier curves stratified by low and high expression of the p-Akt (S473) and p-Akt (T308) proteins by immunohistochemistry. A log-rank test was also performed. In the serous subgroup **(C,D)**, patients with high p-Akt (S473) expression (*n* = 19) had significantly poorer overall survival (OS) than those with low expression (*n* = 99; *P* = 0.008). OS did not differ between patients with high (*n* = 84) and low (*n* = 36) p-Akt (T308) expression (*P* = 0.968). Patients with high p-Akt (S473) expression (*n* = 40) had poorer OS and progression-free survival (PFS) than those with low p-Akt (S473) expression (*n* = 160; *P* = 0.019 and *P* = 0.071, respectively, **E,F**). OS and PFS did not differ between patients with high p-Akt (T308; *n* = 146) and low p-Akt (T308) expression (*n* = 72; *P* = 0.341 and *P* = 0.941, respectively, **G,H**).

Survival analyses of 406 ovarian cancer patients obtained from the TCPA database showed that the protein expression of p-Akt (S473; Figure 1A) but not p-Akt (T308; Figure 1B) correlated with OS of these patients. To add, patients with a higher expression level of p-Akt (S473; *n* = 203) had worse OS compared to patients with lower expression level (*n* = 203; log-rank *p* = 0.002).

### Cellular p-Akt (S473) and p-Akt (T308) Expression in NCKU Patients

Associations between p-Akt expression in EOC tumor tissues at the time of diagnosis and clinicopathological factors were examined. Cellular p-Akt (S473) overexpression was significantly associated with grade 3 tumors (*P* = 0.034), PFI < 6 months (*P* = 0.031), and cancer death (*P* = 0.034; Table 1). However, there was no significant correlation between cellular p-Akt (T308) expression and the analyzed factors (Table 1). Patients with high p-Akt (S473) expression had poorer OS and PFS than those with low p-Akt (S473) expression (*P* = 0.001 and *P* = 0.071, respectively; Figures 1E,G). Besides, OS and PFS did not differ between patients with high and low p-Akt (T308) expression (*P* = 0.341 and *P* = 0.941, respectively; Figures 1F,H).

**TABLE 1:** Patient demographics, immunohistochemistrical expressions of p-Akt (S473) and p-Akt (T308).

		**p-Akt (S473) staining**				**p-Akt (T308) staining**			
			
**Variable**		***N*^1^**	**Low**	**High**	***p***	***N***	**Low**	**High**	***p***
									
**All patients**		**200**	***N* = 160**	***N* = 40**		**218**	***N* = 72**	***N* = 146**	
Age (year)	≤53	105	83 (79.0)	22 (21.0)	0.723	123	42 (34.1)	81 (65.9)	0.689
	>53	95	77 (81.1)	18 (18.9)		95	30 (31.6)	65 (68.4)	
FIGO Stage	Early	68	57 (83.8)	11 (16.2)	0.332	86	27 (31.4)	59 (68.6)	0.679
	Advanced	132	103 (78.0)	29 (22.0)		132	45 (34.1)	87 (65.9)	
Histology	Serous	118	99 (83.9)	19 (16.1)	0.098	120	36 (30.0)	84 (70.0)	0.293
	Non-serous	82	61 (74.4)	21 (25.6)		98	36 (36.7)	62 (63.3)	
Grade	1 and 2	74	65 (87.8)	9 (12.2)	0.034	86	35 (40.7)	51 (59.3)	0.052
	3	126	95 (75.4)	31 (24.6)		132	37 (28.0)	95 (72.0)	
Residual nodules	<1 cm	153	126 (82.4)	27 (17.6)	0.133	171	60 (35.1)	111 (64.9)	0.217
	≥1 cm	47	34 (72.3)	13 (27.7)		47	12 (25.5)	35 (74.5)	
Response to chemotherapy	CR and PR	163	132 (81.0)	31 (19.0)	0.661	157	54 (34.4)	103 (65.6)	0.742
	SD and PD	37	28 (75.7)	9 (24.3)		38	12 (31.6)	26 (68.4)	
PFI	<6 months	53	37 (69.8)	16 (30.2)	0.031	57	24 (42.1)	33 (57.9)	0.090
	≥6 months	147	123 (83.7)	24 (16.3)		161	48 (29.8)	113 (70.2)	
Death	No	105	90 (85.7)	15 (14.3)	0.034	94	27 (28.7)	67 (71.3)	0.239
	Yes	95	70 (73.7)	25 (26.3)		124	45 (36.3)	79 (63.7)	

*^1^With 18 cases with slides failed to be stained by immunohistochemistry. Non-serous histology stained with p-Akt (S473) antibody included mucinous (*n* = 16), endometrioid (*n* = 26), clear cell (*n* = 36), others (*n* = 4). Non-serous histology stained with p-Akt (T308) antibody included mucinous (*n* = 22), endometrioid (*n* = 31), clear cell (*n* = 41), others (*n* = 4). Data were analyzed by X^2^ test or Fisher’s exact test. Abbreviations: FIGO, International Federation of Gynecology and Obstetrics; CR, complete response; PR, partial response; SD, stable disease; PD, progressive disease; PFI, progression-free interval; phospho-Akt (p-Akt).*

In the serous subgroup, cellular p-Akt (S473) overexpression was significantly associated with poor response to chemotherapy (*P* = 0.049) and cancer death (*P* = 0.036) (Supplementary Table S2). OS curves for the 118 patients are presented in Figure 1C. Patients with high p-Akt (S473) expression had poorer OS and PFS than patients with low p-Akt (S473) expression (*P* = 0.008 and *P* = 0.076, respectively). Cellular p-Akt (T308) overexpression was significantly associated with grade 3 tumor (*P* = 0.011; Supplementary Table S2). OS curves for the 120 patients with serous histology are presented in Figure 1D. Both OS and PFS did not differ between patients with high and low p-Akt (T308) expression (*P* = 0.968 and *P* = 0.697, respectively).

Overall, we did not find a statistically significant correlation between patient demographics and percentage of p-Akt (S473 or T308) positive cells, or immunostaining score (intensity × percentage of p-Akt positive cells, range 0–300).

### AZD Compounds Inhibited Akt/mTOR Signaling in Ovarian Cancer Cells, and COL11A1 mRNA and Promoter Activity Was Only Suppressed by AZD5363

**FIGURE 2:**
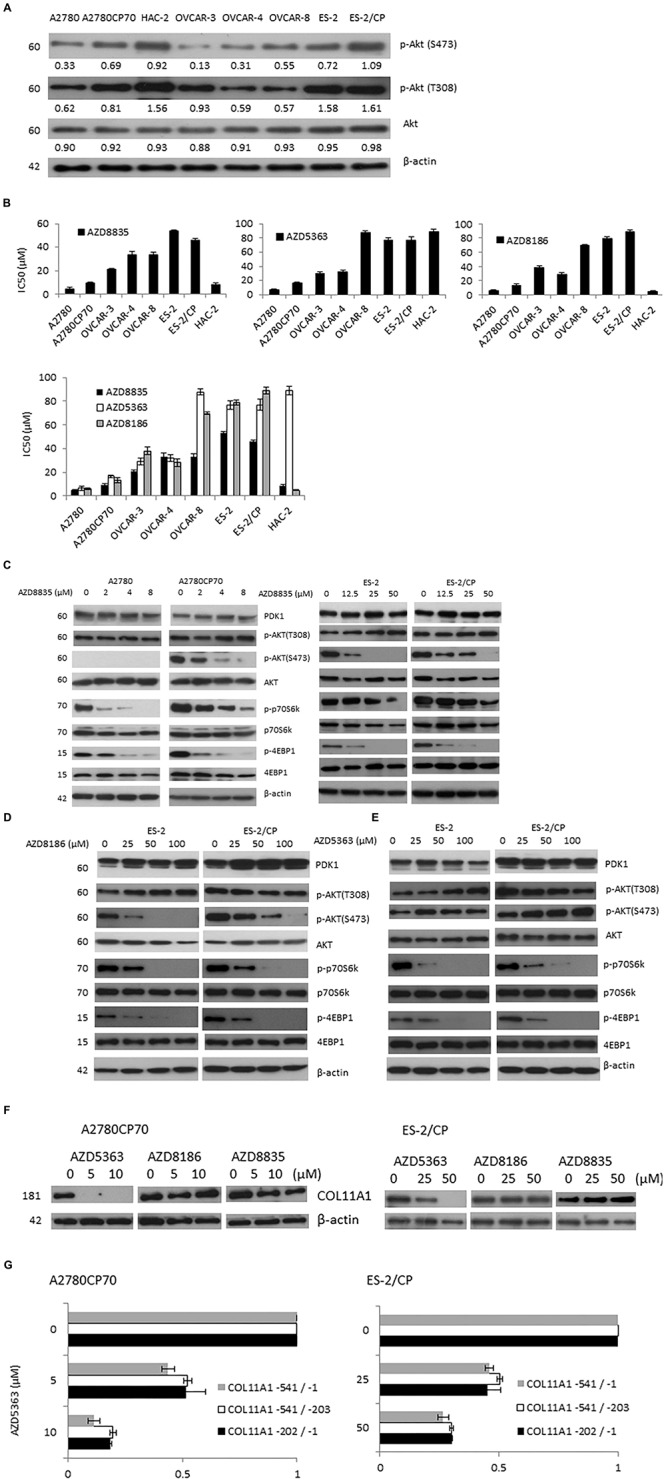
Three AZD compounds inhibit Akt/mTOR signaling in ovarian cancer cells and the mRNA and promoter activity of COL11A1 were only inhibited by AZD5363. **(A)** The protein expression levels of p-Akt (S473), p-Akt (T308), and Akt in a panel of eight ovarian cancer cell lines were evaluated by western blotting. The β-actin protein was used as an internal loading control. All experiments were performed in triplicate. **(B)** The half-maximal inhibitory concentration (IC_50_) value of three AZD compounds in ovarian cancer cells was measured by an MTT assay. **(C–E)** The protein expression levels of PDK1, p-Akt (S473), p-Akt (T308), Akt, p-p70S6K, p70S6K, p-4EBP1, and 4EBP1 in cells treated with different concentrations of AZD compounds for 48 h were evaluated by western blotting. The β-actin protein was used as an internal loading control. All experiments were performed in triplicate. **(F)** The COL11A1 expression in A2780CP70 and ES-2/CP cells treated with different concentrations of AZD compounds for 24 h were evaluated by western blotting. **(G)** A2780CP70 and ES-2/CP cells transfected with the *COL11A1* promoters were treated with different concentrations of AZD5363 for 24 h. Luciferase activity was measured and normalized to Renilla luciferase activity. All experiments were performed in triplicate.

Ovarian cancer is a highly heterogeneous disease, with different histological subtypes and molecular compositions. Thus, a panel of eight ovarian cancer cell lines was tested to derive the expression pattern and activation status of Akt phosphorylation. Cell lysates were prepared without any treatment and baseline western blots were performed. Akt phosphorylation on S473 or T308 was found to be higher in the HAC-2 and cisplatin-resistant cell lines such as A2780CP70 and ES-2/CP cells (Figure 2A).

The IC_50_ values for three AZD compounds were evaluated by the MTT assay. With the exception of low HAC-2 sensitivity to AZD5363 (IC_50_ = 89.14 μM), cells with PI3KCA and/or PTEN mutation (A2780, A2780CP70, and HAC-2 cells) showed greater sensitivity to AZD compounds than cells without mutation (OVCAR-3, OVCAR-4, OVCAR-8, ES-2, and ES-2/CP cells; Figures 2A,B). The IC_50_ values of the PI3KCA mutated cell lines ranged from 4.64 to 9.41 μM for AZD8835 and 4.88 to 13.39 μM for AZD8186, and those with wild-type PI3KCA ranged from 21.14 to 53.60 μM for AZD8835, and 28.16 to 88.71 μM for AZD8186. The IC_50_ values of AZD5363 in A2780 and A2780CP70 cells ranged from 6.46 to 16.61 μM, which are values that differ from the range, 29.28 to 87.78 μM, found for the cell lines with wild-type PI3KCA. OVCAR-8 cells, which have *KRAS* mutation, were relatively insensitive to AZD8186 and AZD5363 compared to OVCAR-3 or OVCAR-4 cells (Figure 2B).

AZD8835 treatment suppressed the expression of p-p70S6K, and p-4EBP1 at lower doses in chemosensitive A2780 (Figure 2C, right panel) and ES-2 (Figure 2C, left panel) cells. To add, AZD8835 treatment was less effective at suppressing the Akt/mTOR pathway in chemoresistant A2780CP70 (Figure 2C, right panel) and ES-2/CP (Figure 2C, left panel) cells. AZD8186 treatment suppressed the expression of p-Akt (S473) at lower doses in chemosensitive ES-2 (Figure 2D) cells and was less effective at reducing the activation of the Akt pathway in chemoresistant ES-2/CP (Figure 2D) cells. Although the p-Akt (S473) expression level was increased, a decrease in the expression level of p-p70S6K and p-4EBP1 was observed with AZD5363 treatment (Figure 2E), indicating the suppression of Akt/mTOR signaling. These findings align with those of Davies et al. (2012). Of note, the three AZD compounds failed to suppress the expression of PDK1 and p-Akt (T308).

Previously, we showed that COL11A1 mRNA expression and promoter activity were regulated by SC66, but not by MK-2206. This finding suggested that Akt inhibitors exert their inhibitory activities through different mechanisms (Wu et al., 2019). Thus, we tested whether these AZD compounds could inhibit COL11A1. Our results showed that the expression level of COL11A1 was inhibited only by AZD5363, but not by AZD8186 and AZD8835 (Figure 2F). In addition, the promoter activity of COL11A1 was decreased after AZD5363 treatment (Figure 2G).

### AZD5363 and AZD8835 Sensitize Ovarian Cancer Cells to Cisplatin and Paclitaxel Therapy

**FIGURE 3:**
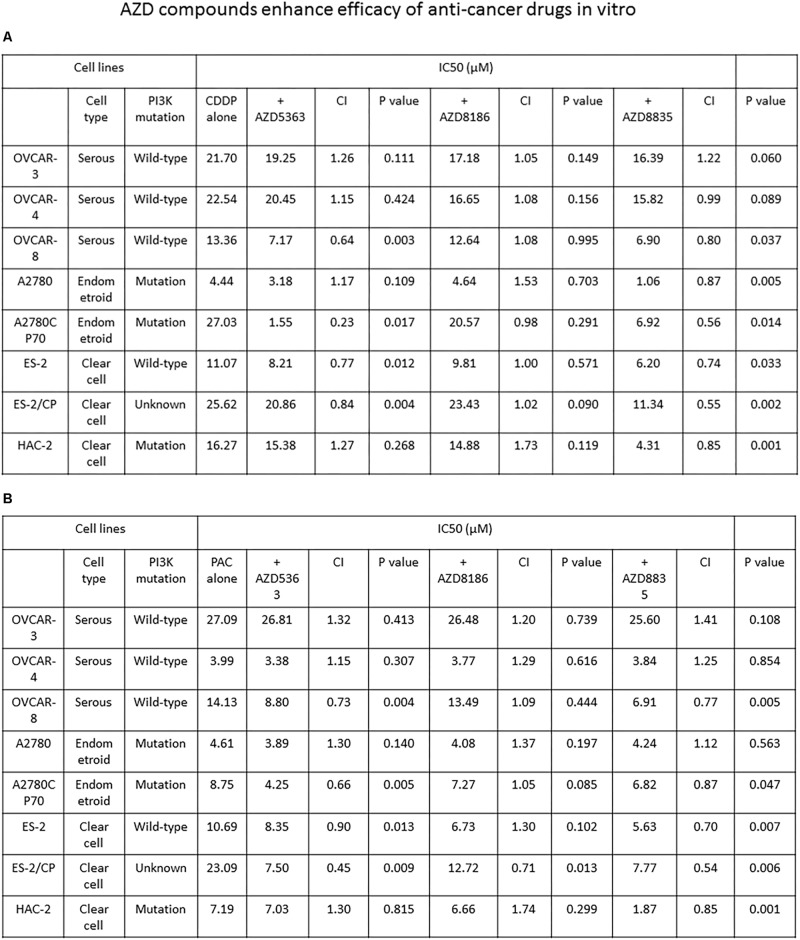
AZD5363 and AZD8835 sensitize ovarian cancer cells to cisplatin and paclitaxel therapy. **(A)** Ovarian cancer cells were treated with different combinations and concentrations of AZD compounds and cisplatin. Each combination was tested with *N* = 5 replicates. After 48 h of treatment, cell viability was assessed by MTT assays. All experiments were performed in triplicate. **(B)** Ovarian cancer cells were treated with different combinations and concentrations of AZD compounds and paclitaxel. Each combination was tested with *N* = 5 replicates. After 48 h of treatment, cell viability was assessed by MTT assays. All experiments were performed in triplicate.

We proceeded to determine whether AZD compounds enhance the efficacy of anticancer drugs. The IC_50_ values of each agent alone or combined, and the CI values of the AZD compounds plus cisplatin or paclitaxel are presented in Figure 3. The CI values of AZD5363 indicate a synergistic cytotoxicity when it is combined with cisplatin or paclitaxel in OVCAR-8 and ES-2 cells. Synergistic cytotoxicity to cisplatin or paclitaxel was also observed in the chemoresistant A2780CP70 and ES-2/CP cells (Figures 3A,B). For AZD8186, the synergistic effect was only observed when AZD8186 was combined with paclitaxel in ES-2/CP cells. To add, the CI values for AZD8835 indicated synergistic cytotoxicity when combined with cisplatin in OVCAR-8, A2780, ES-2, and HAC-2 cells, and the chemoresistant A2780CP70 and ES-2/CP cells. Synergistic cytotoxicity to paclitaxel was observed in OVCAR-8, A2780CP70, ES-2, ES-2/CP, and HAC-2 cells (Figures 3A,B). These results demonstrate that AZD5363 and AZD8835 sensitized chemoresistant cells to treatment with cisplatin and paclitaxel.

### Downregulation of BRCA1 and BRCA2 by AZD8835 in OVCAR-8 Cells

**FIGURE 4:**
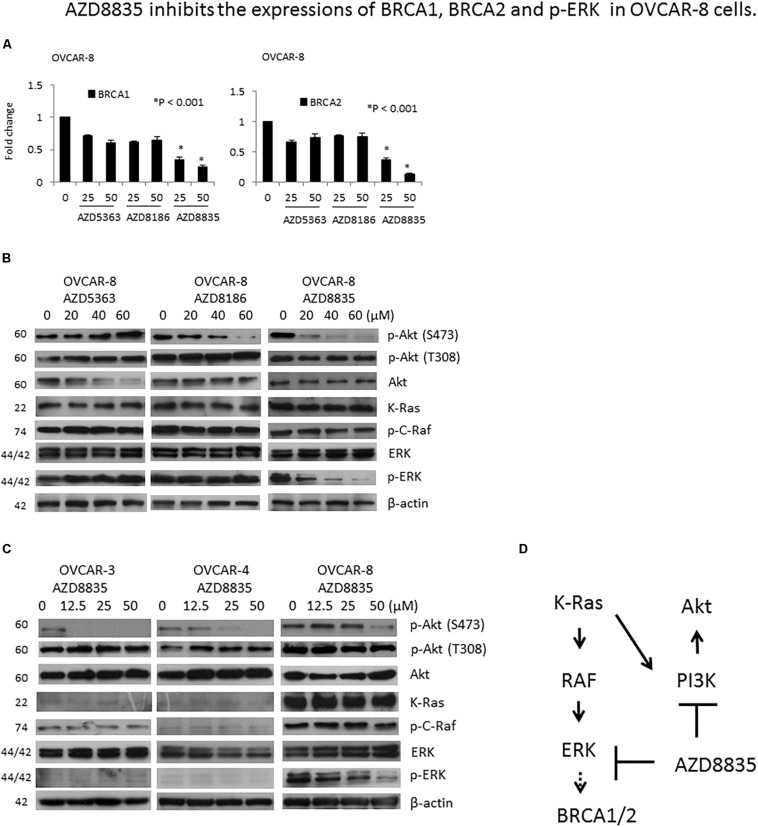
AZD8835 downregulates BRCA1/BRCA2 mRNA expression in *KRAS*-mutated OVCAR-8 cell via ERK signaling. **(A)** The mRNA expression levels of BRCA1 and BRCA2 in OVCAR-8 cells treated with different concentrations of AZD compounds for 24 h were evaluated by real-time RT-PCR. All experiments were performed in triplicate. **(B)** Protein expression levels of p-Akt (S473), p-Akt (T308), Akt, KRAS, p-C-Raf, ERK, and p-ERK in OVCAR-8 cells treated with different concentrations of AZD compounds for 24 h were evaluated by western blotting. The β-actin protein was used as an internal loading control. All experiments were performed in triplicate. **(C)** Protein expression levels of p-Akt (S473), p-Akt (T308), Akt, KRAS, p-C-Raf, ERK, and p-ERK in OVCAR-3, OVCAR-4, and OVCAR-8 cells treated with different concentrations of AZD8835 for 24 h were evaluated by western blotting. The β-actin protein was used as an internal loading control. All experiments were performed in triplicate. **(D)** In the presence of KRAS mutation, AZD8835 inhibited BRCA1/2 expression through ERK signaling.

Previous studies reported that BRCA1/2 downregulation might represent a potential indicator of the treatment response of PI3K inhibition in ovarian cancer cells (Wang et al., 2016). We determined the mRNA expression of BRCA1 and BRCA2 by real-time PCR in OVCAR-8 (Figure 4A), OVCAR-4, HAC-2, and ES-2 cells (Supplementary Figure S2) after AZD compound treatment. Of the cells examined, only OVCAR-8 had a downregulated expression of the BRCA1/2 mRNA after AZD8835 treatment (Figure 4A).

PI3K inhibition may render BRCA-proficient breast tumor cells more responsive to PARP inhibition through ERK signaling-mediated downregulation of BRCA1/2 expression (Ibrahim et al., 2012). As OVCAR-8 is a *KRAS* mutated cell, we tested whether KRAS/ERK signaling is involved in the downregulation of BRCA1/2 in OVCAR-8 cell by AZD8835. Our results showed that of the three AZD compounds, only AZD8835 could inhibit p-ERK expression in OVCAR-8 cells (Figure 4B). The expression levels of KRAS, p-C-Raf, and p-ERK were much higher in OVCAR-8 cells than OVCAR-3 and OVCAR-4 cells, while those of KRAS and p-C-Raf did not change by AZD8835. Besides, AZD8835 decreased p-ERK expression in OVCAR-8 cells (Figure 4C). Our results suggested that in the presence of *KRAS* mutation, AZD8835 inhibits BRCA1/2 expression through ERK signaling (Figure 4D).

### AZD Compounds Inhibit Tumor Growth in Mouse Xenografts

**FIGURE 5:**
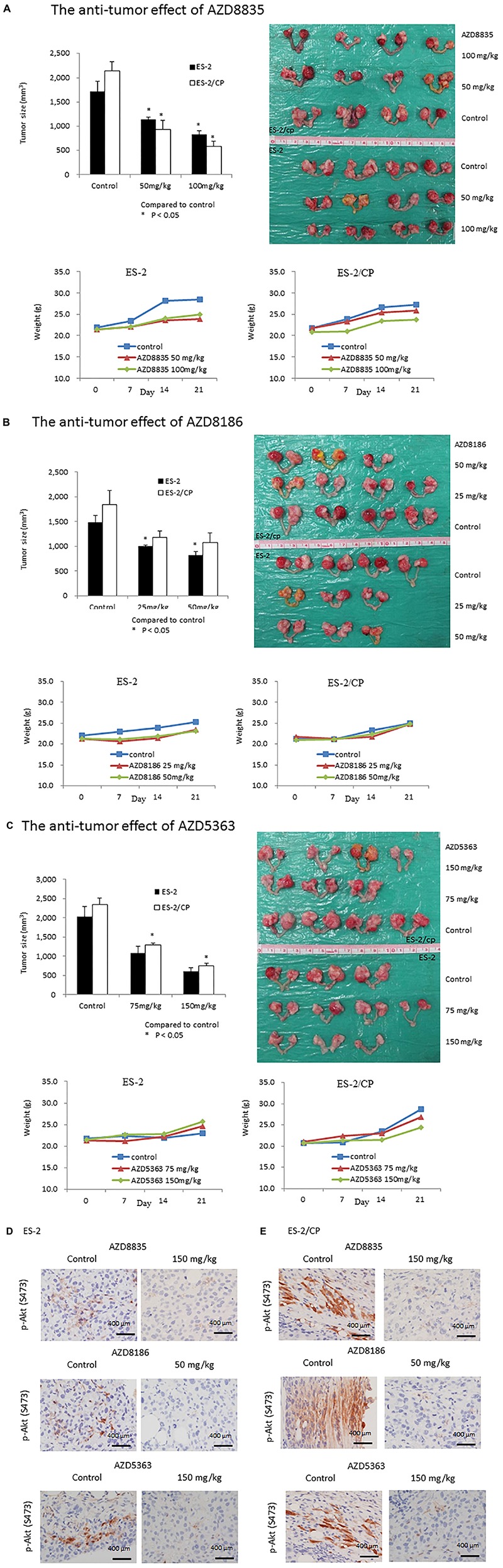
AZD compounds display anti-cancer effects in orthotopic mouse xenografts. **(A)** AZD8835: Tumor inhibition rates in ES2 tumors were 34.1 and 52.2%, whereas those in ES2/CP tumors were 56.3 and 90.4% in mice treated with doses of 50 and 100 mg/kg, respectively. **(B)** AZD8186: Tumor inhibition rates in ES2 tumors were 32.6 and 44.6%, whereas those in ES2/CP tumors were 36.0 and 41.9% in mice treated with doses of 25 and 50 mg/kg, respectively. **(C)** AZD5363: Tumor inhibition rates in ES2 tumors were 46.2 and 43.5%, whereas those in ES2/CP tumors were 44.6 and 41.8% in mice treated with doses of 75 and 150 mg/kg, respectively. **(D,E)** Representative IHC figures of p-Akt (S473) in tumor samples from mice treated with three AZD compounds or vehicle controls. **D:** ES-2; **E:** ES-2/CP. Scale bar: 400 μm.

The antitumor effects of the AZD drugs are illustrated in Figure 5. Compared to the vehicle controls, ES2 tumor was significantly inhibited in mice treated with 50 and 100 mg/kg AZD8835 (*P* = 0.020 and, *P* = 0.020, respectively), or 25 and 50 mg/kg AZD8186 (*P* = 0.030 and *P* = 0.020, respectively; Figures 5A,B). Furthermore, ES2/CP tumor was significantly inhibited in mice treated with 50 and 100 mg/kg AZD8835 (*P* = 0.020 and *P* = 0.020, respectively), or 75 and 150 mg/kg AZD5363 (*P* = 0.030 and *P* = 0.020, respectively; Figures 5A,C). Although the ES2 tumor was inhibited in mice treated with 75 and 150 mg/kg AZD5363 compared to those treated with vehicle, this difference was not statistically significant (*P* = 0.080 and *P* = 0.050, respectively). Similarly, the inhibitory effects in ES2/CP tumor treated with 25 and 50 mg/kg AZD8186 failed to reach statistical significance (*P* = 0.080 and *P* = 0.080, respectively) relative to those treated with vehicle. Body weight of animals remained relatively increased, suggesting a negligible level of toxicity, if any, caused by the treatments (Figure 5, lower left and right panels). IHC confirmed that the expression levels of p-Akt (S473) were significantly decreased by AZD5363, AZD8835, and AZD8186 treatment in mouse xenografts (Figures 5D,E).

## Discussion

Akt activity is frequently dysregulated in cancer and is an important factor in ovarian tumor progression and chemoresistance (Navid and Gerber, 2012). Akt phosphorylation on Ser473 has been extensively studied in tumor samples as a biomarker for Akt activity and clinical outcome, but only limited studies have assessed its effect on Thr308. In the current study, EOC patients with tumor p-Akt (S473) overexpression, but not p-Akt (T308) were found to have shorter PFI, increased cancer death, and shorter OS. Such findings indicated that elevated p-Akt (S473) expression is an unfavorable biomarker of long-term survival. Many Akt inhibitors have been developed and are currently in clinical development (Huck and Mochalkin, 2017). Our findings in the orthotopic animal model demonstrated that AZD compounds, especially AZD8835 and AZD5363, caused a significant reduction in tumor formation. Such *in vivo* findings were reinforced by *in vitro* findings in a panel of eight ovarian cancer cell lines where the three AZD compounds inhibited the Akt downstream effectors, 4E-BP1 and p70S6 kinase. AZD8835 and AZD5363, but not AZD8186 sensitized chemoresistant ovarian cancer cells to cisplatin and paclitaxel treatment. In addition, only AZD5363 could inhibit COL11A1 mRNA and promoter activity. Notably, AZD compounds did not change the mRNA expression of BRCA1/BRCA2 in ovarian cancer cells; however, AZD8835 could downregulate BRCA1/BRCA2 mRNA in *KRAS*-mutated OVCAR-8 cell via ERK signaling.

Overexpressed p-Akt is associated with the prognosis of ovarian cancers (Huang et al., 2011; Tanaka et al., 2011; Jia et al., 2014). A study, however, demonstrated that there is no significant association between p-Akt and OS (Woenckhaus et al., 2007). The significance of Akt phosphorylation on Thr308 remains largely unknown. Vincent et al. (2011) reported that Akt phosphorylation on Thr308 but not on Ser473 correlates with Akt activity in human non-small cell lung cancer. In the current study, our findings showed high p-Akt (S473) expression by the semi-quantification method, IHC, relative to unfavorable survivals in the entire patient cohort and in patients with serous histology. Nonetheless, we did not observe survival differences between patients with low and high p-Akt (T308) expression. Our data regarding p-Akt (S473) expression by IHC are consistent with those from TCPA and TCGA by using a quantitative antibody-based technique, reverse phase protein array (RPPA). To add, there were no survival differences between low and high p-Akt (T308) expression using either IHC or RPPA. Hence, p-Akt (S473) expression, instead of p-Akt (T308), should be used as a prognostic biomarker in EOC.

Herein, we revealed that ovarian cancer cells with *PIK3CA/PTEN* mutations are sensitive to PI3K/Akt inhibitors; this finding is mainly consistent with those obtained in breast or prostate cancer cells (Davies et al., 2012; Hancox et al., 2015; Hudson et al., 2016). A2780 and A2780CP70 cells with *PIK3CA*/*PTEN* mutations were sensitive to the three AZD compounds (Figure 2B). However, although the HAC-2 cell line with *PIK3CA* mutation was sensitive to AZD8835 and AZD8186, it was resistant to AZD5363 (Figure 2B). Measuring mutation status or activation status of additional genes, such as *KRAS*, provides a potential marker of resistance in tumors with aberrant or deregulated PIK3CA/PI3Kα (Weigelt and Downward, 2012). *PIK3CA* mutant breast cancer cell lines, with additional mutations in *KRAS*, are shown to confer resistance to inhibition by AZD8835 (Hudson et al., 2016). Colon and bladder cancer cells with *KRAS* mutation are resistant to AZD5363, although accompanied by coincident PIK3CA mutations (Davies et al., 2012). Consistently, our results showed that the OVCAR-8 cell line with *KRAS* mutation and wild-type *PIK3CA* exhibited resistance to AZD5363 relative to other OVCAR-derived cells with wild-type KRAS and PIK3CA (Figure 2B). Further investigation is required to examine whether KRAS mutation is present in HAC-2 cells.

Previous report indicated that the expressions of p-Akt (S473) and p-Akt (T308) were inhibited by AZD8835 in breast cancer cells (Hudson et al., 2016). In this study, treatment with AZD8835 and AZD8186 did not change the expression of p-Akt (T308; Figures 2C,D). This might be due to the unchanged expression of PDK1, a kinase well known to be responsible for the phosphorylation of Akt on Thr308 (Wu et al., 2019), post-treatment with the AZD compounds (Figures 2C,D).

The level of BRCA1/2 expression was reported to be downregulated by the PI3K inhibitor, BKM120, in wild-type *PIK3CA* ovarian cancer cells (OVCA433, OVCAR-5, and OVCAR-8), without the involvement of ERK activation (Wang et al., 2016). In this study, PI3K inhibition by AZD8835 led to a decrease in phosphorylated ERK signals and BRCA1/2 mRNA in wild-type PIK3CA, but not in KRAS mutated OVCAR-8 cell (Figures 5B,C). Our results provide the novel finding that in the presence of KRAS mutation, AZD8835 inhibits BRCA1/2 expression through ERK signaling. A further investigation is required to explore the exact mechanisms of ERK-dependent BRCA1/2 expression by AZD8835.

The present study indicated that of the three AZD compounds, only AZD5363 could inhibit COL11A1 mRNA and promoter activity, and none could change the expression of PDK1 (Figure 2E). Our previous report indicated that COL11A1 enhances cell sensitivity to anti-cancer drugs via the activation of the Akt/c/EBPβ pathway in concert with attenuated PDK1 ubiquitination and degradation (Wu et al., 2015). These results might explain why chemo-resistant cells with high COL11A1 expression tended to show greater synergistic effect to AZD5363 and anti-cancer drugs (Figures 3A,B). Previously, we showed that COL11A1 mRNA expression and promoter activity were regulated by SC66, but not by MK-2206 (Wu et al., 2019). SC66 has been shown to promote Akt ubiquitination (Wu et al., 2019), whereas AZD5363 inhibits Akt downstream function (Davies et al., 2012). These findings suggest that PDK1 could phosphorylate Akt even with the decrease of COL11A1 by the Akt inhibitor. Previously, we revealed that COL11A1 is an important factor in Akt regulation and chemoresistance in ovarian cancer via its activation of the Akt/COL11A1/c/EBP pathway and reduction of PDK1 degradation (Wu et al., 2015). PDK1 is involved in the signaling pathways that frequently altered in cancers, such as PI3K/Akt, Ras/MAPK, and Myc. Together, our findings indicate that most Akt inhibitors could not inhibit COL11A1 and PDK1 should alter the readers that the selection of appropriate PI3K/Akt inhibitors may be crucial for future clinical development.

To our knowledge, clinical trials that use the three AZD drugs tested herein for gynecologic cancers are rare. Preliminary results of a phase II trial revealed longer PFS and OS when AZD5363 was added to first-line taxane therapy for metastatic triple-negative breast cancer (Schmid et al., 2018). A RECIST response of 8% was observed in 56% of PI3KCA-mutated gynecologic patients treated with AZD5363 (480 mg bid, 4/7 intermittent dose) (Banerji et al., 2018). Furthermore, a phase I trial of AZD8835 for advanced solid tumor was completed but report of its findings is not available^3^ (#NCT02260661). To add, a phase I trial of AZD8186 and docetaxel for PTEN or PIK3CB mutated solid tumor is ongoing. Nonetheless, our results provide clinical and dose-dependent implications of AZD5363 or AZD8835 in platinum-resistant ovarian cancer. Further well-designed investigations should, however, be performed in the clinic.

This study has few limitations such as the discordant results regarding *in vitro* versus *in vivo* antitumor effects of AZD compounds in combination with paclitaxel. Another limitation is the lack of animal experiments combining AZD compounds with chemotherapy drugs to support our *in vitro* synergy findings. Subsequently, the expression levels of p-Akt (S473), PDK1, and COL11A1 could be determined in tumors.

## Data Availability Statement

All datasets generated for this study are included in the article/Supplementary Material.

## Ethics Statement

The studies involving human participants were reviewed and approved by the National Cheng Kung University Hospital Institutional Review Board (A-ER-105-017) and written consent was obtained from each patient. Our study complied with the National Centre for the Replacement, Refinement and Reduction of Animals in Research, and was approved by the Institutional Animal Care and Use Committee of National Cheng Kung University.

## Author Contributions

Y-HW designed and performed the experiments. Y-FH analyzed the data and discussed the results. C-CC conducted the IHC experiments. C-YH analyzed the TCPA and TCGA data. Y-HW, Y-FH, and C-YC drafted the manuscript. All authors edited and approved the final manuscript.

## Conflict of Interest

The authors declare that the research was conducted in the absence of any commercial or financial relationships that could be construed as a potential conflict of interest.

## Footnotes

1 http://www.tcpaportal.org/tcpa/index.html

2 http://cancergenome.nih.gov/

3 ClinicalTrials.gov
