# Supplementary material for: Comparing PI3K/Akt Inhibitors Used in Ovarian Cancer Treatment
Source: Front Pharmacol. 2020 Mar 3;11:206. doi: 10.3389/fphar.2020.00206 (PMC7063971; doi:10.3389/fphar.2020.00206)
Supplement: Supplementary file 1 [file Data_Sheet_1.docx]

**Comparing PI3K/Akt inhibitors used in ovarian cancer treatment**

Yi-Hui Wu^1^, Yu-Fang Huang^2^, Chien-Chin Chen^3,4^, Chia-Yen Huang^5,6,7^ and Cheng-Yang Chou^2^

**Supplementary Table 1. Patient demographics, expressions of p-Akt (S473), p-Akt (T308) and Akt in TCGA dataset.**

|  | |  | | **p-Akt (S473)** | | | | | | **p-Akt (T308)** | |  | |  | **Akt** | | | |  |
| --- | --- | --- | --- | --- | --- | --- | --- | --- | --- | --- | --- | --- | --- | --- | --- | --- | --- | --- | --- |
| Variable | | | | N | Low | High | *p* |  | Low | | High | | *p* |  | Low | | High | *p* | |
|  | | | |  | N=204 (%) | N=204 (%) |  |  | N=204 (%) | | N=204 (%) | |  |  | N=204 (%) | | N=204 (%) |  | |
| Age (year)^1^ | ≤ 58 | | 206 | | 113 (54.9) | 93 (45.1) | 0.060 |  | 105 (51.0) | | 101 (49.0) | | 0.730 |  | 96 (46.6) | 110 (53.4) | | 0.150 | |
|  | > 58 | | 201 | | 91 (45.3) | 110 (54.7) |  |  | 99 (49.3) | | 102 (50.7) | |  |  | 108 (53.7) | 93 (46.3) | |  | |
| FIGO Stage^2^ | Early | | 34 | | 24 (70.6) | 10 (29.4) | **0.014** |  | 17 (50.0) | | 17 (50.0) | | 0.960 |  | 24 (70.6) | 10 (29.4) | | **0.010** | |
|  | Advanced | | 369 | | 179 (48.5) | 190 (51.5) |  |  | 186 (50.4) | | 183 (49.6) | |  |  | 177 (48.0) | 192 (52.0) | |  | |
| Residual nodules^3^ | < 1 cm | | 257 | | 138 (53.7) | 119 (46.3) | **0.004** |  | 126 (49.0) | | 131 (51.0) | | 0.520 |  | 124 (48.2) | 133 (51.8) | | 0.530 | |
|  | ≥ 1 cm | | 110 | | 41 (37.3) | 69 (62.7) |  |  | 58 (52.7) | | 52 (47.3) | |  |  | 57 (51.8) | 53 (48.2) | |  | |
| Response to chemotherapy | CR & PR | | 234 | | 124 (53.0) | 110 (47.0) | **< 10^-5^** |  | 121 (51.7) | | 113 (48.3) | | 0.120 |  | 114 (48.7) | 120 (51.3) | | 0.700 | |
|  | SD & PD | | 46 | | 8 (17.4) | 38 (82.6) |  |  | 18 (39.1) | | 28 (60.9) | |  |  | 21 (45.7) | 25 (54.3) | |  | |
| Death^4^ | No | | 162 | | 98 (60.5) | 64 (39.5) | **< 0.001** |  | 81 (50.0) | | 81 (50.0) | | 0.970 |  | 87 (53.7) | 75 (46.3) | | 0.270 | |
|  | Yes | | 241 | | 104 (43.2) | 137 (56.8) |  |  | 121 (50.2) | | 120 (49.8) | |  |  | 116 (48.1) | 125 (51.9) | |  | |

1 with 1 missing data

2 with 5 missing data

3 with 41 missing data

4 with 5 missing data

Data was analyzed by X^2^ test or Fisher’s exact test.

Abbreviation: FIGO, International Federation of Gynecology and Obstetrics; CR, complete response; PR, partial response; SD, stable disease; PD, progressive disease.

**Supplementary Table 2. Patient demographics, immunohistochemistrical expressions of p-Akt (S473) and p-Akt (T308) in serous subgroup in NCKUH.**

|  |  | | | p-Akt (S473) staining | |  |  |  | p-Akt (T308) staining | | |  |
| --- | --- | --- | --- | --- | --- | --- | --- | --- | --- | --- | --- | --- |
| Variable | | | N^1^ | Low | High | *p* |  | N | | Low | High | *p* |
| **Serous subgroup** | | | 118 | N=99 | N=19 |  |  | 120 | | N=36 | N=84 |  |
| Age (year) | | ≤ 53 | 55 | 44 (80.0) | 11 (20.0) | 0.282 |  | 59 | | 17 (28.8) | 42 (71.2) | 0.780 |
|  |  | > 53 | 63 | 55 (87.3) | 8 (12.7) |  |  | 61 | | 19 (31.1) | 42 (68.9) |  |
| FIGO Stage | | Early | 19 | 17 (89.5) | 2 (10.5) | 0.470 |  | 22 | | 6 (27.3) | 16 (72.7) | 0.757 |
|  |  | Advanced | 99 | 82 (82.8) | 17 (17.2) |  |  | 98 | | 30 (30.6) | 68 (69.4) |  |
| Grade | | 1 & 2 | 47 | 42 (89.4) | 5 (10.6) | 0.189 |  | 49 | | 21 (42.9) | 28 (57.1) | 0.011 |
|  |  | 3 | 71 | 57 (80.3) | 14 (19.7) |  |  | 71 | | 15 (21.1) | 56 (78.9) |  |
| Residual nodules | | < 1 cm | 78 | 69 (88.5) | 9 (11.5) | 0.060 |  | 82 | | 26 (31.7) | 56 (68.3) | 0.549 |
|  |  | ≥ 1 cm | 40 | 30 (75.0) | 10 (25.0) |  |  | 38 | | 10 (26.3) | 28 (73.7) |  |
| Response to chemotherapy | | CR & PR | 93 | 81 (87.1) | 12 (12.9) | 0.049 |  | 86 | | 27 (31.4) | 59 (68.6) | 0.476 |
|  |  | SD & PD | 25 | 18 (72.0) | 7 (28.0) |  |  | 25 | | 6 (24.0) | 19 (76.0) |  |
| PFI | | < 6 months | 36 | 28 (77.8) | 8 (22.2) | 0.231 |  | 38 | | 14 (36.8) | 24 (63.2) | 0.266 |
|  |  | ≥ 6 months | 82 | 71 (86.6) | 11 (13.4) |  |  | 82 | | 22 (26.8) | 60 (73.2) |  |
| Death | | No | 57 | 52 (91.2) | 5 (8.8) | 0.036 |  | 41 | | 12 (29.3) | 29 (70.7) | 0.900 |
|  |  | Yes | 61 | 47 (77.0) | 14 (23.0) |  |  | 79 | | 24 (30.4) | 55 (69.6) |  |

^1^ with 2 cases with slides failed to be stained by immunohistochemistry.

Data was analyzed by X^2^ test or Fisher’s exact test.

Abbreviation: FIGO, International Federation of Gynecology and Obstetrics; CR, complete response; PR, partial response; SD, stable disease; PD, progressive disease; PFI, progression-free interval; phospho-Akt (p-Akt)

**Supplementary Figure 1. Protein expression levels of p-Akt (S473) and p-Akt (T308) by immunohistochemistry.**

**Supplementary Figure 2. The mRNA expression levels of BRCA1 and BRCA2 in OVCAR-4, HAC-2, and ES-2 cells treated with different concentrations of AZD compounds for 24 h were evaluated by real-time RT-PCR. All experiments were performed in triplicate.**
