# Supplementary material for: Comparing PI3K/Akt Inhibitors Used in Ovarian Cancer Treatment
Source: Front Pharmacol. 2020 Mar 3;11:206. doi: 10.3389/fphar.2020.00206 (PMC7063971; doi:10.3389/fphar.2020.00206)
Supplement: Supplementary file 2 [file Presentation_1.PPT]

## Slide 1
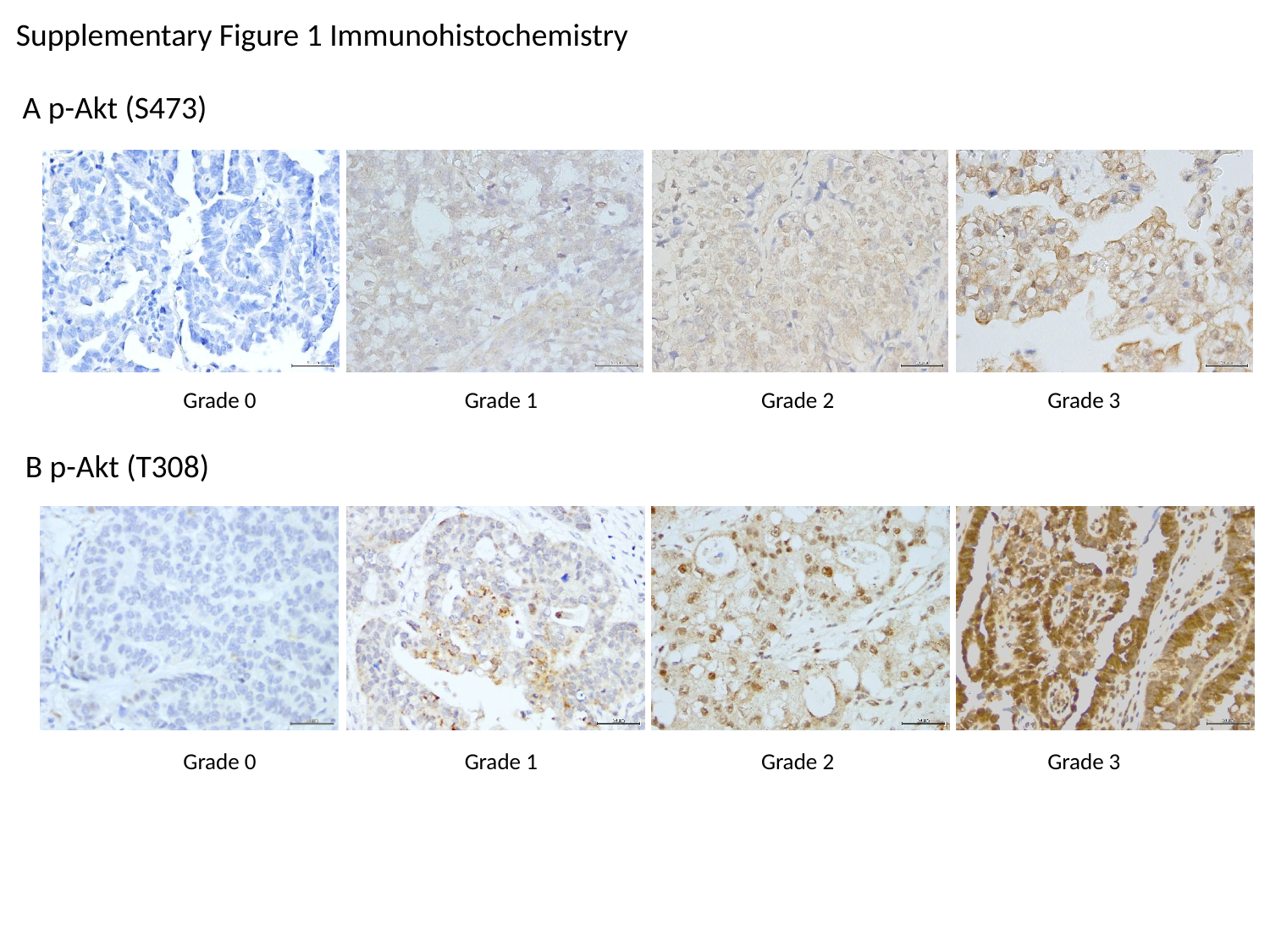

Supplementary Figure 1 Immunohistochemistry
A p-Akt (S473)
Grade 0 Grade 1 Grade 2 Grade 3
B p-Akt (T308)
Grade 0 Grade 1 Grade 2 Grade 3

## Slide 2
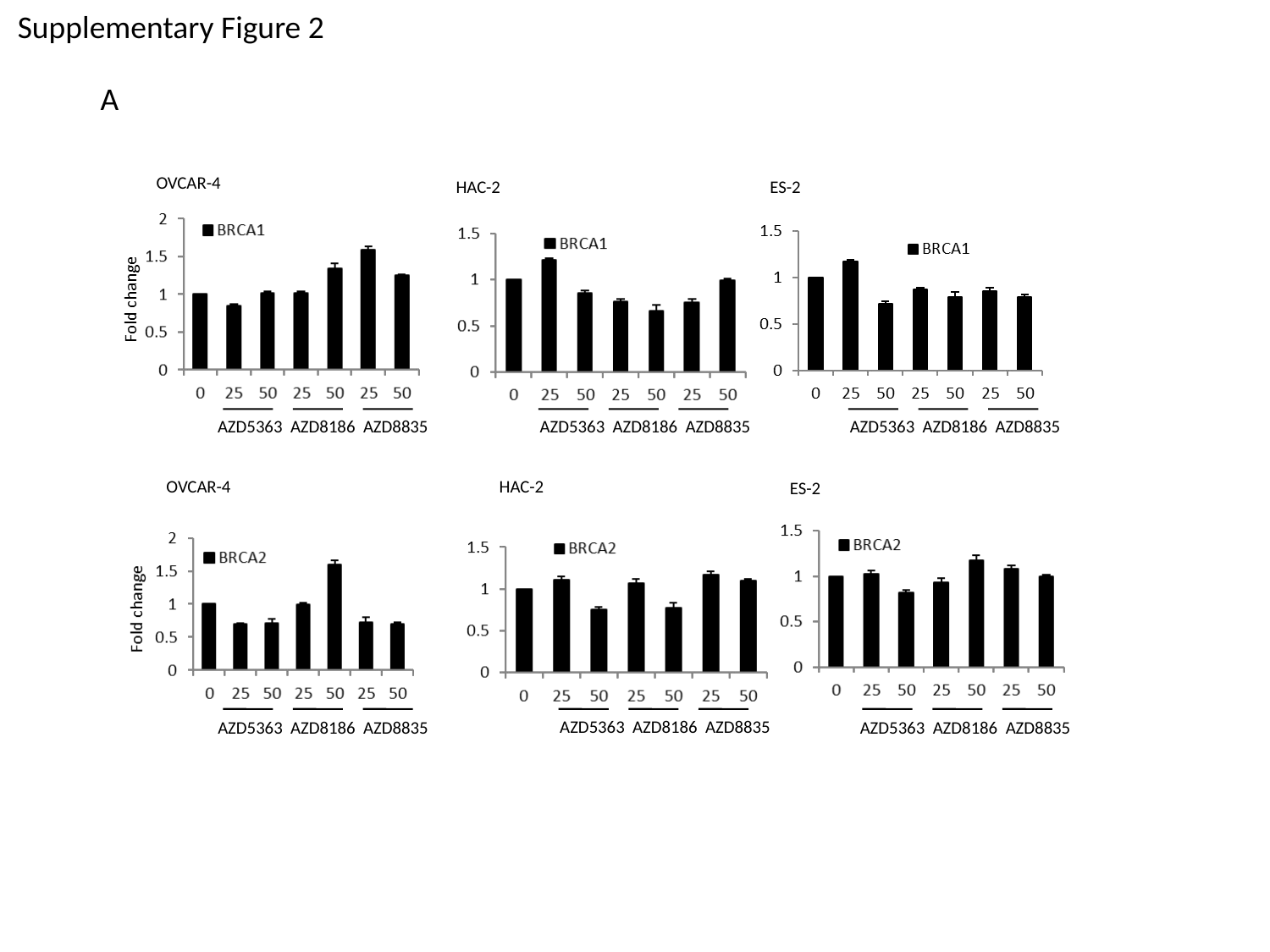

Supplementary Figure 2
A
OVCAR-4
HAC-2
ES-2
AZD5363 AZD8186 AZD8835
AZD5363 AZD8186 AZD8835
AZD5363 AZD8186 AZD8835
HAC-2
OVCAR-4
ES-2
AZD5363 AZD8186 AZD8835
AZD5363 AZD8186 AZD8835
AZD5363 AZD8186 AZD8835
